# One Rotten Apple Infects All in the Basket

**DOI:** 10.3201/eid1901.AC1901

**Published:** 2013-01

**Authors:** Polyxeni Potter

**Affiliations:** Author affiliation: Centers for Disease Control and Prevention, Atlanta, Georgia, USA

**Keywords:** art science connection, emerging infectious disease, art and medicine, Still Life with Two Figures, Pieter Cornelisz van Rijck, food, food-borne infections, listeriosis, about the cover

**Figure:**
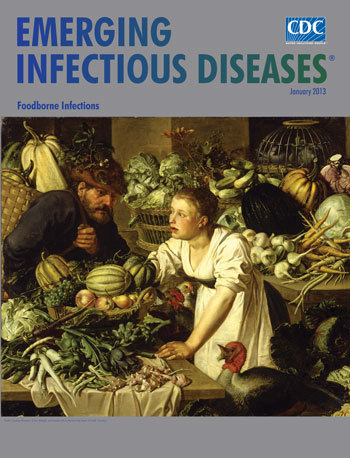
**Pieter Cornelisz van Rijck (1558‒1628) *Still Life with Two Figures* (1622) Oil on canvas (123.8 cm × 148.6 cm)** North Carolina Museum of Art, Raleigh, purchased with funds from the State of North Carolina

Food was a precious commodity during the Dutch Golden Age. Along with industry, trade, and wealth, gastronomy flourished in the Netherlands during the late 16th and well into the 17th century. We know because, among other sources, art of the region, an extraordinary output in a short time span, is filled with luscious images of banquets. The wealthy had access to the best ingredients from all over the world, and city dwellers had an advantage over rural folk. Even the poor saw better diets, if only from a modest improvement in work and living conditions and an increase in charity. This was, too, a golden age for art lovers. Art acquisition, a rich person’s sport, was more prevalent at this time, with burghers, the biggest group of urban residents, becoming affluent enough to purchase art. This was “anonymous” art―not commissioned but produced for the mass market and tied not to the name of any one artist but to the value of the individual work in the eyes of the buyer.

“The Dutch,” wrote Daniel Defoe in A Plan of the English Commerce (1728), “must be understood as they really are, the Middle Persons in Trade, the Factors and Brokers of Europe… they *buy* again to *sell* again, *take* in to *send* out, and the greatest Part of their vast Commerce consists in being supply’d from All Parts of the World that they may supply All the World again.” Defoe’s interest was in the talent of the Dutch to buy and sell to advantage, but in terms of food, this extraordinary global exchange, celebrated and meticulously documented in art, foreshadowed and rivaled food commerce in our times.

During the Golden Age, northern Netherlands provinces were predominantly rural, unlike the southern, which were urban. Agriculture and fishing formed the basis of the economy, and animal husbandry ruled. Because the soil in many provinces did not favor wheat farming, livestock husbandry became the predominant form of agriculture. Grazing grounds found their way into art, but the farmers and their monotonous existence not so much, even though farmers were potent symbols of honest work and frugal living. Frequently featured livestock included cattle, goats, sheep, and chickens. Moral messages abounded, usually promoting hard work as a prerequisite to material wealth. Fishing, a traditional means of livelihood, expanded with technology, spreading outside the local market to become the very foundation of international trade. A frequent feature in art images, the herring became a symbol of Dutch prosperity, an example of “how great things grew from humble beginnings,” although the fishermen themselves, at the bottom of the social ladder, rarely were central figures.

As urbanization spread, many farmers turned to market gardening to satisfy expanding demand for vegetables. This interaction of urban and agricultural sectors, an integral part of society, was reflected in art, both in the selection of topics and the style of execution. Vegetables played a big role in Dutch diet. They were brought into towns by barges and small boats from outlying farm areas to be sold at city markets. They were also shipped from France, Italy, and Germany. Even more than fishermen and livestock farmers, horticulture farmers were focused on the market, with the prices depending on the ease of cultivation and seasonal availability.

Hardy fruits and vegetables were available year-round at low prices and made up the diet of the urban poor. These were also popular in still life paintings: apples, onions, squashes, turnips, artichokes, cabbages, and orange Horn carrots―which represented Dutch innovation in breeding new varieties. For the affluent, having pictures of these vegetables, or even eating them from time to time, served as reminder of the virtues of modest life. More elaborate and exotic vegetables, such as asparagus, also were featured, even if rarely eaten. Only the affluent could afford difficult crops and imports; yet images of opulent tables and extravagant fare, as in the works of Frans Snyders, Nicolaes Gillis, Pieter Claesz, and many others, also appealed to those with little access to such feasts.

Open air markets in major cities were gathering places. “Every morning, the wives of farmers and fishermen, though occasionally the men as well, would enter the cities to sell their goods…. People had incentives to form personal relationships, vendors wanting to maintain regular customers and buyers hoping for discounts.” Urban markets were guided by city regulations that dictated hours of operation and vendor behavior. Separate markets existed for vegetables and fruits, fish, saltwater fish, and poultry, though not all municipalities could afford appropriate space and facilities or standards of cleanliness. In general, damaged vegetables were not allowed, and fish markets operated only in the morning before the sun could rot the fish. Some vendors, bakers for instance, had a clear advantage. Demand for their product was so high that they were prosperous enough to commission portraits.

Marketplace depictions were not idealized in art. Apart from occasionally placing exotic products in the wrong context or mixing fresh-water with salt-water fish, food was not altered. A bruised or damaged vegetable or fruit was an opportunity for a moral lesson. Poor quality questioned the integrity of the vendor, reminding the customer that one bad apple could damage the lot and favoring vendors who used stringent standards. And, of course, aging or damage held lessons about the ephemeral nature of all things.

Reconstructing the food history of a place from topical images is always challenging, even when the images come from a master of kitchen interiors and market scenes. Pieter Cornelisz van Rijck was one of the “important contemporary painters” listed by Karel van Mander, artist biographer and chronicler of the Dutch Golden Age. He encouraged local artists to travel abroad and learn from the world’s heritage. Pieter Cornelisz van Rijck followed the advice. A student in his early days of Jacob Willemsz Delff, he later studied with Huybrecht Jacobsz Grimani, whom he accompanied to Italy, spending considerable time in Naples, where he continued to study and work. There he also came under the influence of Jacopo Bassano.

Pieter Cornelisz van Rijck’s *Still Life with Two Figures* is a snap-shot of the food genre, a close-up of abundance and prosperity as well as the energy with which food was moved and promoted. The two figures capture unnamed farm workers in the midst of marketplace bustle. The man, a large basket strapped on his back, brings the goods to the stall. He is alert and purposeful. The woman in charge of this vibrant scene has stout arms and clear eyes. The produce is firm and fresh, and caged poultry hint at the range of ways by which one could make a living from the farm. The large birds in the foreground complement the colorful mess.

Market scenes around the world have not changed much, though trade of perishable foods and the urgency to move them from farm to table have changed. Dutch market scenes, the one by Cornelisz van Rijck among them, showed a sense of urgency. Perishables were intended to move as quickly as possible because blemishes and spoilage posed economic risks. Modern food technologies, such as refrigeration, permit a longer travel time. But as refrigerated perishable goods get manipulated en route, new risks are introduced. Pathogens are moving into new food items, modifying and expanding the nature and epidemiologic characteristics of foodborne disease. Despite the differences in emphasis from spoilage to microbiological contamination that causes disease outbreaks, the old rotten apple emblem still applies. Mishandled food from a single manufacturer can still spoil the lot as it raises havoc with the marketplace.
